# Post-COVID lung injury and the need for longitudinal histology

**DOI:** 10.3325/cmj.2025.66.460

**Published:** 2025-12

**Authors:** Barina Khan

**Affiliations:** Karachi Medical and Dental College, Karachi, Pakistan

To the Editor,

I read with interest the recent article by Salai et al (1), who examined histopathological findings in patients with persistent respiratory symptoms following COVID-19 pneumonia. The authors are to be commended for performing transbronchial lung biopsies in a complex clinical setting and for offering a tentative pathological timeline of lung injury progression.

That said, several points merit consideration. First, while the paper suggests a chronological pattern of injury, the cross-sectional design makes it difficult to definitively establish temporal progression. Barisione et al have shown through matched radiologic and histopathologic analyses that fibrotic progression in COVID-19 lungs is best understood through serial data (2). Second, the male predominance in the study (91%) may limit generalizability. Sex-based differences in immune and fibrotic responses have been observed in earlier post-COVID cohorts and could influence histologic patterns (3). Third, while the authors acknowledge limitations of forceps biopsy, cryobiopsy has demonstrated superior diagnostic yield and deeper sampling in interstitial lung disease, including in COVID-related conditions (4). Lastly, the presence of rare findings such as fibrosing non-specific interstitial pneumonia and lepidic carcinoma highlights the importance of differential diagnosis. As Cha et al noted, chronic COVID-related lung damage may mimic or coexist with other pathologies (5). In sum, this work adds valuable insight into long COVID pathology. Future studies using multicenter prospective designs and cryobiopsy protocols may help clarify disease trajectories and better define clinical subtypes.
